# Une compression médullaire révélant une hydatidose vertébro-médullaire

**DOI:** 10.11604/pamj.2013.16.113.3096

**Published:** 2013-11-23

**Authors:** Toufik Joulali, Mohammed Khatouf

**Affiliations:** 1 Service de Réanimation Polyvalente, CHU Hassan II, Fès, Maroc

**Keywords:** compression médullaire, hydatidose, paraparésie, spinal cord compression, hydatidosis, paraparesis

## Image en médicine

Enfant âgé de 12 ans, sans antécédents pathologiques notables, est admis aux urgences pour une paraparésie évoluant depuis 23 jours. L'interrogatoire a révélé la présence de troubles sphinctériens et l'examen clinique a mis en évidence des reflexes ostéo-tendineux abolis avec un tonus flasque et un déficit sensitivomoteur bilatéral. L'imagerie par résonnance magnétique a montré la présence d'images de vésicules hydatiques au niveau de D11-D12 avec lyse osseuse du corps vertébral et compression médullaire. Le patient a bénéficié d'une laminectomie D11-D12, avec évacuation des kystes, curetage prudent et ostéosynthèse L1-D10. L’évolution a été marquée par une récupération totale de la force musculaire au niveau des membres inférieurs. La localisation vertébrale du kyste hydatique est de mauvais pronostic et reste exceptionnelle: 0,5 à 2,5% de l'ensemble des localisations hydatiques. Sa symptomatologie clinique est non spécifique avec une évolution lentemais inéluctable vers la compression médullaire ou radiculaire. Le diagnostic repose sur un faisceau d'arguments épidémiologiques, cliniques, biologiques et surtout radiologiques grâce aux moyens modernes d'imagerie. Le traitement reste essentiellement chirurgical, avec un pronostic défavorable et un taux de récidive pouvant atteindre les 40%.

**Figure 1:**
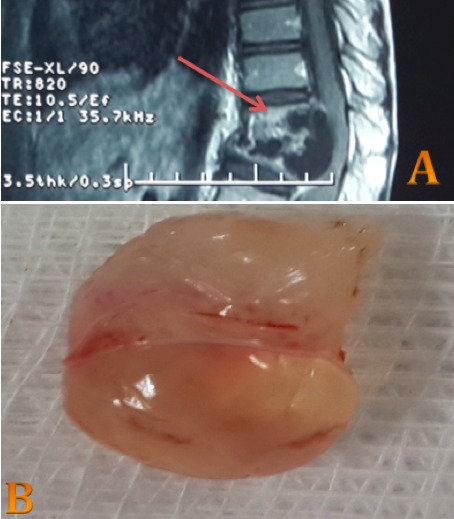
A) IRM en coupe sagittale T2 objectivant un processus multi vésiculaire vertébro-médullaire avec extension canalaire; B): produit de l’évacuation médullaire: vésicule hydatique

